# Evaluation of an electrostatic precipitator in mitigating the transmission of airborne viruses in experimentally infected pigs

**DOI:** 10.1186/s13567-025-01503-1

**Published:** 2025-04-04

**Authors:** Lan Wang, José Morán, My Yang, Bernard A. Olson, Christopher J. Hogan, Montserrat Torremorell

**Affiliations:** 1 Department of Veterinary Population Medicine, College of Veterinary Medicine, University of Minnesota, St. Paul, MN USA; 2 Department of Mechanical Engineering, College of Science and Engineering, University of Minnesota, Minneapolis, MN USA

**Keywords:** Electrostatic precipitator, airborne virus transmission, influenza, PRRS, pigs, air control technology

## Abstract

Airborne viruses spread rapidly in animal premises, which makes them difficult to contain. Electrostatic precipitators (ESP) are air cleaning devices that charge airborne particles and electrophoretically deposit them on collection surfaces, thereby removing them from an airstream. We evaluated the effect of a single-stage wire-plate ESP on mitigating airborne transmission of influenza A virus (IAV) and porcine reproductive and respiratory syndrome virus (PRRSV), using experimentally infected pigs. Inoculated pigs were placed in isolators upstream of the ESP, and sentinel pigs were placed downstream in an isolator. The airflow moved unidirectionally from inoculated pigs to sentinel pigs. Nasal swabs of pigs, air samples and surface wipes from all the isolators were collected daily and tested by RT-qPCR. Without the ESP powered, sentinel pigs tested positive within 1 day of exposure to IAV aerosols and 2 days to PRRSV aerosols. Airborne IAV RNA was detected upstream and downstream of the ESP in particles ranging from 0.22 μm to > 8 μm. In contrast, with the ESP powered, sentinel pigs tested positive after 5–6 days of exposure to IAV aerosols, and 7–8 days to PRRSV aerosols. Limited levels of IAV RNA were detected in air samples in the downstream isolator before sentinel pigs tested positive. The RNA-based virus removal efficiency of the ESP ranged from 96.91 to 99.97%, with higher removal observed in particles > 6.5 μm. Under the conditions of this study, the ESP efficiently removed IAV aerosol particles and delayed the onset of IAV and PRRSV infections in the sentinel pigs. Our study shows the potential of the ESPs to help prevent the spread of airborne viruses in agricultural animal farming facilities.

## Introduction

Airborne diseases are costly and difficult to contain and control in today’s agricultural settings. This is especially relevant for domestic animals, such as swine and poultry reared in confined and controlled ventilated environments where commonly exhaust fans help transport particles to the outside. Diseases affecting swine including porcine reproductive and respiratory syndrome (PRRS), swine influenza and porcine epidemic diarrhea (PED) can be transmitted through aerosols [1–3] and lead to significant economic losses in the swine industry. PRRSV is one of the most devastating diseases in swine, characterized by reproductive failure in breeding sows and respiratory disorders in pigs of any age. In the United States, PRRS was estimated to cause losses of almost $664 million per year from 2006 to 2010 [4]. A PRRSV variant of lineage 1C 1-4-4 appeared in late 2020 and resulted in large-scale PRRS outbreaks in the Midwestern US [5]. This variant was found to be of higher infectivity and potential transmissibility compared to other PRRSV-2 lineage 1 strains [6, 7]. PRRSV was reported in air inside the barns [8] and downwind, at distances up to 4.7 km and 9.1 km from barns with PRRSV inoculated pigs [3, 9]. Influenza A virus (IAV) is an important respiratory pathogen of swine that causes significant production losses up to $10/pig due to high morbidity, variable mortality and increased susceptibility to secondary infections [10–12]. IAV also poses a constant threat to public health due to its potential to generate new variants by reassortment of viral genomes derived from strains of various species, such as the 2009 H1N1 pandemic strain. IAV has been detected in air samples collected inside barns, in exhausted air and downwind at up to 2.1 km from infected farms [2].

Regional airborne transmission of PRRSV has driven swine producers to invest in air filtration systems to prevent the introduction of PRRSV into herds [13]. Although filters can be effective at removing airborne pathogens from air intake and reducing incidence of PRRSV infections in large breeding herds [14], there are several shortcomings of utilizing intake filtration as the only measure to mitigate airborne disease transmission. The pressure drop brought about by filters leads to increased pumping requirements and increased energy consumption for the ventilation systems, in addition to the large costs associated with purchase, installation and replacement of filters. Besides, filters only provide a means to collect particles but do not incorporate an inactivation mechanism, leading to a risk of resuspension of viable virus-containing particles. Therefore, there is a need to develop new technologies and protocols capable of effectively controlling the spread of infectious aerosols and preventing airborne disease introduction and regional dissemination [15].

Airborne viruses are usually incorporated into particles of various sizes [16]. The size of the virus-laden particles largely determines the behavior of the particles including how far the particles travel, how long they stay suspended in the air, where they deposit in the respiratory tract if inhaled, and the viral load and infectivity [17–19]. There are air control technologies available to remove and/or inactivate virus-laden particles. Of particular interest are electrostatic precipitators (ESPs), which are a competitive technology with filtration that could be utilized towards improved biosecurity against airborne pathogens. ESPs are air cleaning devices that utilize corona discharge needles or wires to generate ions; these ions charge particles via ion-particle collisions, and subsequently particles electrophoretically migrate to grounded collection electrodes. ESPs can have high removal efficiencies of > 99% in collecting particles [20] across an extremely wide size range, from 10 nm to more than 10 μm. For this reason, they have been widely used in large scale coal combustion plants and in residential HVAC systems for collection of dust and soot by-products of combustion reactions [21–24]. Importantly, ESPs have extremely low pressure drops, comparable to that of common HVAC ductwork, and do not need frequent replacement, which should result in lower cost and energy consumption compared to air filtration systems. While high voltages are required for ESP operation, the requisite power, which is the product of the corona discharge ionization current and the applied voltage, is typically small in comparison to the product of the air flow rate and pressure drop across filter, which defines the increased fluid power associated with filter implementation. Furthermore, ESPs can potentially inactivate viruses as the intense electric field at the ESP wire surface leads to production of reactive oxygen species [25–28] which are effective in inactivating viruses via oxidation reactions [29].

There have already been demonstration studies showing that ESPs and related technologies can remove and inactivate artificial infectious (i.e. nebulized) aerosols [26, 28] when operating at high voltages (~ tens of kV), and that ESPs are able to remove virus-laden particles of IAV, PRRSV and PEDV of different sizes generated by infected pigs [30]. However, ESPs have not been evaluated directly in mitigation of airborne transmission from one animal to another. Such evaluation requires addressing several experimental challenges associated with virus aerosol transmission studies with animals [31–33], including the need to isolate animals while permitting aerosol exposure, to sample the low viral concentration of animal generated aerosols (compared to intentional generation in a laboratory), and to characterize real respiratory aerosol size distributions from animals. In this study, we overcame these challenges by using animal isolator chambers connected with air-permeable ducts and sampling size-classified airborne particles with Andersen cascade impactors, and evaluated the ability of a single-stage ESP to mitigate airborne transmission of IAV and PRRSV using experimentally infected pigs. In the experiments, we installed an ESP between isolator chambers; two upstream chambers housing 4 inoculated pigs with either IAV or PRRSV, and one downstream chamber housing 2 sentinel (uninoculated) pigs. Air samples were collected in the upstream and downstream chambers using Andersen cascade impactors, enabling the analysis of RT-qPCR and virus titration for size-classified airborne virus-laden particles generated by the pigs. Air samples and surface samples, alongside nasal swab samples from pigs were collected over multiple days to monitor airborne virus concentrations and the progression of infection in the inoculated and sentinel pigs, both with and without the ESP powered. Results are not only used to estimate the removal efficiency of viruses by the ESP, but provide convincing evidence that IAV and PRRSV are readily transmitted through aerosols.

## Materials and methods

### Transmission experiment setup

**Figure 1:**
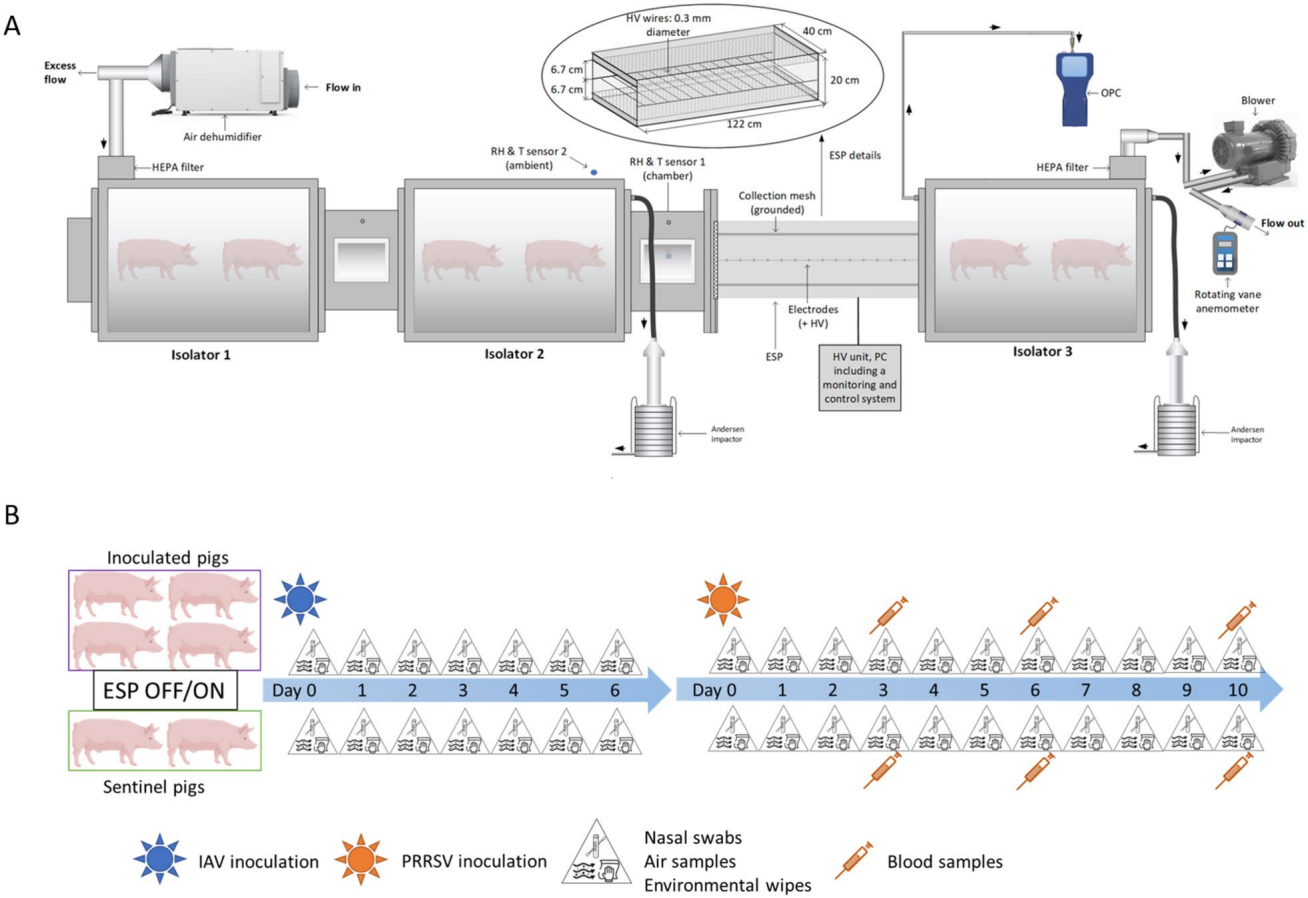
**A schematic diagram of the experimental design**. (**A**) Animal modular isolator system. Two isolators were located upstream of the electrostatic precipitator (ESP) and one isolator downstream. The location of Andersen impactor sampling probes and the optical particle counter (OPC) are also shown. (**B**) Pig inoculation and sampling schedule.

A modular animal isolator system specifically designed to house animals under BSL-2 conditions was used to evaluate the airborne transmission of two viruses using pigs as a model (Figure 1A). The system consisted of three self-contained isolator chambers (volume ~ 0.5 m^3^) built of stainless steel and plexiglass materials operating under negative pressure conditions. Isolators 1 and 2 were connected through a 43.18 cm long and rectangular cross-section duct. The design of the in-house ESP has been described in detail elsewhere [34] as well as its physical collection efficiency and ability to charge particles previously [35]. The ESP consisted of a total of 9 Tungsten wires of 40 cm length, 0.3 mm diameter, equally spaced along the ESP length of 122 cm and located at 6.7 cm from the collecting plates [26]. Briefly, when a high voltage (~ tens of kV) is applied to a sharp metal as the wires of this ESP, a corona discharge is generated. This phenomenon involves the generation of unipolar ions traveling from the high voltage electrodes to the grounded mesh (under positive polarity). These ions collide with particles resulting in the latter being electrically charged and thus electrophoretically collected on the grounded mesh under electric fields. The ESP together with another rectangular cross-section duct was installed between isolator 2 and isolator 3. Each duct contained two metallic grids, approximately fitting the rectangular cross section; the grids permitted unidirectional air flow with negligible pressure drop from isolator 1 to isolator 2 and then to isolator 3, but prevented nose-to-nose contact between animals in the different isolators. Air samples were collected upstream and downstream of the ESP using two Andersen cascade impactors (ACI). An optical particle counter was connected to the isolator 3. These sampling devices are described in “Sampling procedures” sect.

We tested the effect of the ESP with pigs inoculated with an influenza A virus (IAV) and a porcine reproductive and respiratory syndrome virus (PRRSV). For each virus, we conducted a positive control test with the ESP turned off and 3 tests with the ESP turned on (2 tests with the ESP operating at 12 kV and 1 test at 14 kV). The higher voltage yields significantly more ions to charge particles, and a slightly stronger electric field to facilitate electrophoretic particle collection [35]. Four pigs were inoculated with one of the viruses and placed upstream of the ESP in isolators 1 and 2 (two pigs per isolator). Downstream of the ESP, in isolator 3, there were two uninoculated pigs serving as sentinels. The air flow inside the system was set at 850 L min^−1^, which was generated by a blower (Cyclonair Blower, Model CHE 3, Rotron Inc.) with a voltage control unit (VOAG) connected to the air outlet in isolator 3. The isolator system had an approximate volume of 1.79 m^3^, leading to 28.5 air changes per hour during testing. The system was operated under negative pressure with the air moving unidirectionally from the inoculated to the sentinel pigs. The incoming and exhaust air of the system was filtered using HEPA filters. A dehumidifier was connected to the air inlet of isolator 1 to keep the relative humidity (RH) inside the system within 40% to 60%. The temperature inside the system was kept around 25.6–26.7 °C by the heaters in each isolator. Feeders and waterers were available in each isolator, ensuring the pigs had free access to food and water throughout the duration of the study.

The ESP central wires were connected to a high-voltage power supply unit Matsusada model AU-30R1-LC (+/− 30 kV, 1 mA) operated in positive polarity. The corona current and voltage generated on these wires were continuously monitored and recorded by a monitoring and control system. Two relative humidity and temperature sensors were placed inside the connecting duct of the isolators and in the BSL-2 room housing the isolators, respectively. A metal mesh filter (MERV 4) was installed in front of the inlet of the ESP, serving as a prefilter to remove coarse particles.

### Virus growth

Influenza A virus (A/swine/Minnesota/PAH-618/2011 (H1N1)) was grown and titrated in Madin-Darby canine kidney (MDCK) cells. MDCK cells were obtained from the University of Minnesota Veterinary Diagnostic Laboratory (VDL). The cells were purchased from the American Type Culture Collection (ATCC) reference number ATCC-CCL-34. MDCK cells were cultured in Dulbecco’s Modified Eagle Medium (DMEM) supplemented with 10% fetal bovine serum (Life Technologies, Grand Island, NY, USA), 1% antibiotic–antimycotic solution (100X solution containing penicillin, streptomycin, and Gibco Amphotericin B; Life Technologies), 10 mM HEPES (N-2-hydroxyethylpiperazine-N-2-ethane sulfonic acid; Life Technologies), and 1 mM sodium pyruvate (Life Technologies). IAV was propagated in MDCK monolayers with IAV growth media, composed of DMEM supplemented with 4% of 7.5% bovine serum albumin fraction V (Life Technologies), 1.5 µg/mL trypsin-TPCK (N-tosyl-L-phenylalanine chloromethyl ketone treated trypsin), 1% antibiotic–antimycotic solution, 50 µg/mL gentamicin (Life Technologies), and 50 µg/mL neomycin (Teknova, Hollister, CA, USA) at 37 °C with 5% CO_2_ until cytopathic effects (CPE) appeared, usually within 3–5 days.

PRRSV L1C 1-4-4 variant, a newly emerged pathogenic PRRSV variant causing significant losses to the swine industry [5], was grown and titrated in derived monkey kidney cells (MARC-145). MARC-145 cells were obtained from the University of Minnesota Veterinary Diagnostic Laboratory (VDL). The cells were purchased from the National Animal Disease Center (NADC lot number 1511). The MARC-145 cells were cultured in minimal essential medium (MEM) supplemented with 8% fetal bovine serum, and 1% antibiotic–antimycotic solution as described above. PRRSV was propagated in MARC-145 monolayers with PRRSV growth media, composed of MEM supplemented with 4% fetal bovine serum and 1% antibiotic–antimycotic solution at 37 °C with 5% CO_2_ until CPE appeared, usually within 5–7 days. The viruses were titrated using the TCID_50_ method in specific cells, as described in Qiao et al. [36]), and frozen at −80 °C until used.

### Animal infection

A total of 24, eighteen to twenty-one-day-old weaned pigs of either sex (6 pigs in each test) were purchased from a high health closed herd (Midwest Research Swine, LLC, MN, USA), clinically-free and serologically negative for IAV and PRRSV and other important swine pathogens such as *Actinobacillus pleuropneumoniae* and *Mycoplasma hyopneumoniae*. Pigs were sampled by collecting blood and nasal swabs on arrival at the isolation units and confirmed negative for IAV and PRRSV by RT-PCR. Serum samples of all pigs tested negative for IAV and PRRSV antibodies using commercially available ELISA tests (IDEXX Swine Influenza Virus Ab Test and PRRS X3 Ab Test, Westbrook, ME, USA). In each test, two out of six pigs (sentinels) were moved into the downstream isolator 72 h after arrival, and the remaining four pigs were inoculated intranasally with 2 mL (1 mL per nostril) of 10^5.75^ TCID_50_/mL H1N1 influenza A virus inoculum and placed into the two upstream isolators (two pigs per isolator). One week post IAV inoculation, the four upstream pigs were inoculated intranasally with 2 mL (1 mL per nostril) of 10^5.5^–10^6.5^ TCID_50_/mL PRRSV L1C 1-4-4. Clinical signs were recorded for all pigs daily throughout the duration of the study. All pigs were euthanized using an overdose of a pentobarbital solution once PRRSV infection was confirmed in the sentinel pigs or ten days post PRRSV inoculation, whichever came first.

### Sampling procedures

Pigs were manually restrained and removed from the isolator for daily sampling. In order to prevent cross-contamination between groups, the sentinel pigs from the downstream isolator were sampled first. The feeder and waterer were then refilled and the waste inside the isolator was cleaned. After placing the sentinel pigs back in their isolator and locking the door, we repeated the procedures for the inoculated pigs. Personal protective equipment (PPE) including N95 masks, disposable gloves, hair nets, coveralls, goggles, face shields and disposable gowns were worn by the researchers handling the pigs. Researchers changed gloves and gowns each time after handling pigs from the previous group and before proceeding to the next group. Tools used for feed and water were designated for each isolator and decontaminated every day. Sentinel and inoculated pigs were sampled daily by collecting nasal swabs (BBL CultureSwab™ liquid, Stuart single plastic applicator; Becton, Dickinson and Co. Sparks, MD) from 0 to 6 days post IAV inoculation and 0 to 10 days post PRRSV inoculation. In the case of PRRSV, pigs were also bled at 3, 6 and 10 days. The experimental design is shown in Figure 1B.

To measure the concentrations of airborne IAV and PRRSV and the virus-laden particle size distribution, air samples were collected daily from the upstream and downstream isolators using two non-viable Anderson cascade impactors (ACI) operating simultaneously. The ACI is an air sampler capable of collecting airborne particles and separating them by size in up to 9 size intervals ranging from 0.22 μm to > 8 μm when operating at 90 L min^−1^ and stages −2 to 5 employed [37]. Air samples were collected for 30 min in duplicate each day. After collection, samples were eluted directly from each ACI collection plate using a cell scraper and 3 mL of virus transport media, composed of DMEM supplemented with 2% of 7.5% bovine serum albumin fraction V, 1.5 µg/mL trypsin-TPCK, 1% antibiotic–antimycotic and 50 µg/mL gentamicin. The filter on the final ACI stage was eluted with 15 mL of virus transport media. The sizes and concentrations of the total airborne particles were measured downstream using an optical particle counter (AeroTrak Handheld Airborne Particle Counter, Model 9306, TSI Inc., St. Paul, MN), which reported particle number concentrations in 6 size intervals ranging from 0.3 μm to > 10 μm. Surface samples were collected daily using wipes, which were prepared with 3 × 3 inches sterile gauzes moistened with 10 mL of transport media. Samples were collected by wiping the surfaces of the feeder and waterer inside each isolator, and the interior of the downstream isolator at high areas out of pigs’ reach. After collection, all samples were placed on ice and transported to the laboratory for processing and testing.

### Sample processing and testing

Nasal swabs were suspended in 2 mL of virus transport media, vortexed for 15 s, and the suspension was divided into two aliquots. Each blood sample was centrifuged at 1500 × *g*, 4 °C for 10 min, and the serum was pipetted out with a sterile transfer pipette and divided into two aliquots. Each wipe was squeezed in its individual bag, and the suspension was pipetted with a sterile transfer pipette into two aliquots. For all the samples including air samples, one aliquot was placed at 4 °C for testing within 48 h after collection and the other aliquot was stored at –80 °C for later use.

Viral RNA was extracted from 50 μL of each sample using the MagMAX^™^-96 Viral RNA Isolation Kit (Applied Biosystems by Thermo Fisher Scientific, CA, USA) according to the manufacturer’s instructions with a semi-automatic MagMAX Express-96 Deepwell Magnetic Particle Processor (Applied Biosystems by Thermo Fisher Scientific). The RNA was eluted with 50 μL of elution buffer and quantified by quantitative RT-PCR using Ag Path-ID One Step RT-PCR Kit (Applied Biosystems, Foster City, CA, USA). For IAV RT-qPCR, primers and probe targeting the IAV matrix gene [38] were used; for PRRSV RT-qPCR, primers and probe targeting ORF-6 gene were used [39]. The components of the RT-qPCR reaction mixture (25 μL) and the thermocycling conditions for PRRSV and IAV are described in Nirmala et al. [39]. The absolute viral genome copy numbers in each sample, expressed as RNA copies/mL, were quantified using the cycle threshold (Ct) values and standard curves constructed using serial ten-fold dilutions of transcript RNA specific for each virus with known concentrations. In each RT-qPCR run, no template control was used as negative control. Samples with cycle threshold (Ct) value ≤ 35 were considered positive.

For the PCR positive air samples, virus isolation was performed using cells specific for each virus (see sect. “Virus growth”). First, 100 μL or 200 μL of each sample were diluted to 250 μL respectively, with the appropriate virus growth media, and 250 μL of each sample dilution were inoculated into two wells containing cell monolayers in 6-well plates and incubated at 37 °C with 5% CO_2_ for 1 h. Then, 2 mL of virus growth media were added to each cell well and incubated at 37 °C with 5% CO_2_. Cytopathic effects were recorded at 5 days post-inoculation for IAV, and 7 days post-inoculation for PRRSV.

### Data analysis

Viral RNA load (genome copies/m^3^ of air) collected from the various stages of the ACI were normalized by dividing by the width of the size interval on a logarithmic scale. The effect of the ESP on virus-laden particle removal at different size ranges was reported as log reduction, defined as the base 10 logarithm of the ratio of the average airborne viral RNA concentration upstream to the average airborne viral RNA concentration downstream, in reference to all the days before sentinel pigs tested positive. A value of 50 copies/m^3^ was given to samples below the limit of detection of IAV PCR for purpose of calculation. To determine if the log reduction of viral RNA concentration differed significantly among different size ranges, Kruskal–Wallis test was conducted using R [40]. Multiple comparisons of means were performed according to post-hoc Dunn's test and the significance level was corrected using the Bonferroni method.

## Results

### Quantification of virus nasal shedding

**Figure 2:**
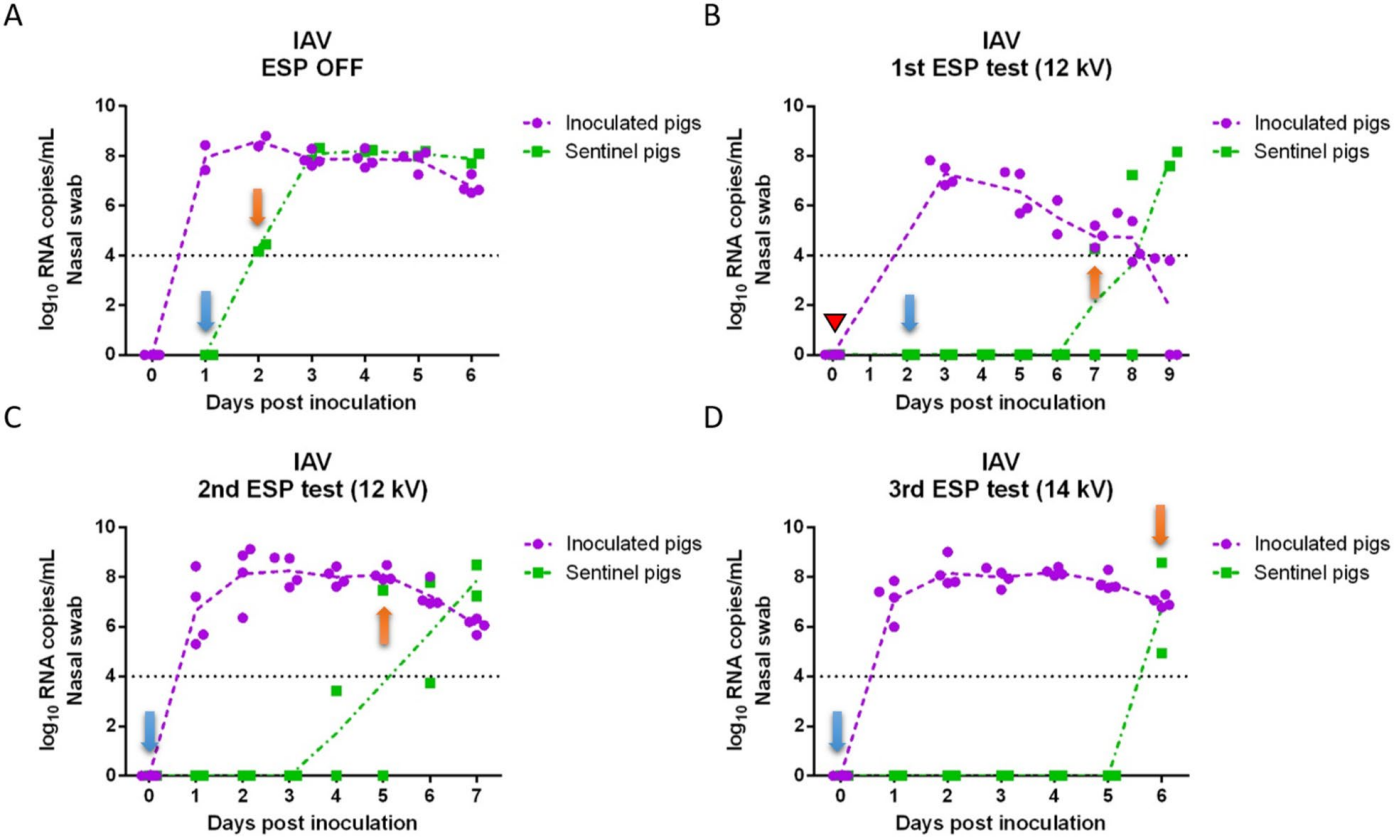
**Influenza A viral RNA concentration in nasal swabs collected daily from inoculated and sentinel pigs**. (**A**) ESP was off (positive control test); (**B**) ESP operated at 12 kV (1^st^ test); (**C**) ESP operated at 12 kV (2^nd^ test); and (**D**) ESP operated at 14 kV (3^rd^ test). A red triangle indicates that the ESP was malfunctioning and the isolators were disconnected from the system. Blue arrows indicate the days when sentinel pigs were placed into the downstream isolator; orange arrows indicate the first day when at least one sentinel pig tested positive.

**Figure 3:**
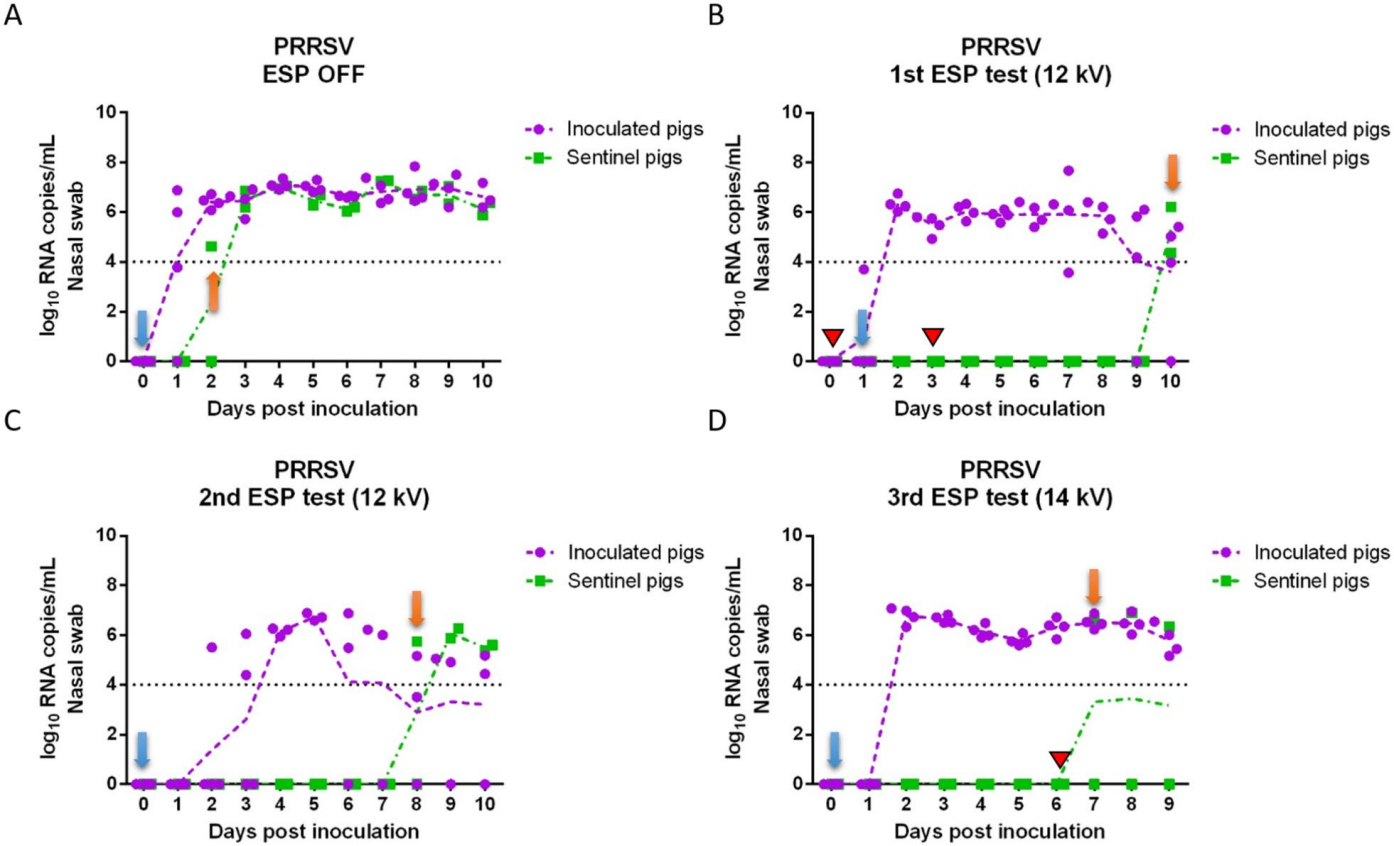
**Porcine reproductive and respiratory syndrome viral RNA concentration in nasal swabs collected daily from inoculated and sentinel pigs**. (**A**) ESP was off (positive control test); (**B**) ESP operated at 12 kV (1^st^ test); (**C**) ESP operated at 12 kV (2^nd^ test); (**D**) ESP operated at 14 kV (3^rd^ test). Red triangles indicate days that the ESP was malfunctioning, and the isolators had to be disconnected briefly (~1 day) during the 1^st^ ESP test, and that the ESP was shut down accidentally during the 3^rd^ test (~4 h). Blue arrows indicate the days when sentinel pigs were placed into the downstream isolator; orange arrows indicate the first day when at least one sentinel pig tested positive.

Viral RNA copy concentrations in nasal swabs collected daily from inoculated and sentinel pigs for each of the tests are shown in Figure 2 (IAV) and Figure 3 (PRRSV). IAV inoculated pigs shed IAV continuously from 1 day post-inoculation (dpi) to 6 dpi (Figures 2A–D) with peak of shedding at 2 dpi (8.24 log_10_ RNA copies/mL on average). In the positive control test (ESP off), sentinel pigs were placed into the downstream isolator at 1 dpi and tested IAV positive at 2 dpi (Figure 2A), which indicated that IAV transmitted through the air within 24 h after infection of the inoculated pigs. For the remaining tests with the ESP on, sentinel pigs were placed in the downstream isolator at 2 dpi (1^st^ test) or 0 dpi (2^nd^ and 3^rd^ tests) prior to the inoculation of pigs upstream. The delay of placing sentinel pigs in the first test was due to a malfunction of the ESP which was resolved by 2 dpi. In the first ESP test (Figure 2B), sentinel pigs were exposed to the aerosol from inoculated pigs at 2 dpi, which resulted in one of the sentinel pigs testing IAV positive at 7 dpi (total exposure time until testing positive was 5 days). Similarly, in the second and third ESP test (Figures 2C and D), sentinel pigs tested IAV positive on 5 dpi and 6 dpi, respectively. The ESP working at 14 kV (3^rd^ test) resulted in one more day in delaying the infection of sentinel pigs compared to those at 12 kV.

In the case of PRRSV, most of the PRRSV-inoculated pigs shed virus continuously from 2 to 10 dpi (Figures 3A–D), and the concentration of viral RNA in nasal swabs ranged between 3.5 and 7.8 log_10_ RNA copies/mL. During the positive control test (ESP off), one sentinel pig tested PRRSV positive at 2 dpi (Figure 3A). In the first and second ESP tests at 12 kV (Figures 3B and C), sentinel pigs tested positive at 10 dpi and 8 dpi respectively, after being exposed with ESP-treated aerosols for 8 days. In the third ESP test at 14 kV (Figure 3D), one of the sentinel pigs tested positive on 7 dpi.

### Viral load and size distribution of airborne virus-laden particles

Total airborne IAV RNA copy concentration of air samples collected daily can be seen in Table 1. The total concentrations are determined by summing the RNA copy concentration measured by each ACI stage. During the IAV positive control test (ESP off), viral RNA was detected in the upstream isolators as early as 1 dpi (4.9 × 10^4^ RNA copies/m^3^) and daily throughout 6 dpi. The peak of airborne RNA concentration was at 2 dpi (6.1 × 10^5^ RNA copies/m^3^). Airborne RNA concentration from the downstream isolator was similar to that from the upstream isolators before sentinel pigs tested positive at 2 dpi, which indicated that IAV transmitted through the air efficiently. In the three ESP tests (ESP on), the concentration of airborne IAV RNA in the upstream isolator was similar to that of the positive control test. Before the sentinel pigs tested positive, viruses detected in the downstream isolator originated from inoculated pigs upstream. However, before the sentinel pigs tested positive, the average viral RNA concentration in the downstream isolator was much lower than that in the upstream isolators with an estimated log reduction ranging from 1.51 to 3.48, corresponding to an overall removal efficiency of 96.91% to > 99.97%, indicating that the ESP was effective at removing viruses from the airflow.

**Table 1:** **Total concentration of influenza A viral RNA collected daily by Andersen cascade impactors upstream and downstream of the electrostatic precipitator and estimated log reduction of concentrations before sentinel pigs tested positive**

		Airborne RNA copies/m^3^					
		1 dpi	2 dpi	3 dpi	4 dpi	5 dpi	6 dpi
ESP OFF	Upstream	4.9E + 04	6.1E + 05	1.6E + 05	4.9E + 04	4.3E + 04	2.2E + 04
	Downstream	4.0E + 04	3.1E + 05	9.5E + 04	1.3E + 05	2.1E + 05	6.1E + 04
	Log reduction	0.10	NA	NA	NA	NA	NA
ESP 12 kV (1^st^ test)	Upstream	NC	1.4E + 05	1.8E + 04	9.1E + 04	7.4E + 03	1.2E + 03
	Downstream	NC	1.3E + 03	ND	2.8E + 03	ND	ND
	Log reduction	NA	2.02	2.56	1.51	2.17	1.38
ESP 12 kV (2^nd^ test)	Upstream	2.2E + 04	2.8E + 05	1.0E + 05	1.2E + 05	5.0E + 05	5.0E + 04
	Downstream	ND	3.1E + 03	2.3E + 03	5.4E + 03	3.4E + 04	3.9E + 03
	Log reduction	2.64	1.96	1.65	NA	NA	NA
ESP 14 kV (3^rd^ test)	Upstream	1.1E + 04	2.8E + 05	8.7E + 04	1.5E + 05	7.6E + 04	3.5E + 03
	Downstream	1.5E + 04	3.3E + 03	ND	ND	ND	3.4E + 04
	Log reduction	−0.16	1.92	3.24	3.48	3.18	NA

NC: not collected.

ND: not detected. A value of 50 copies/m^3^ was given to ND samples for purpose of calculation.

NA: not applicable. Log reduction was not applicable to estimate the ESP removal efficiency after sentinel pigs tested positive.

Data show the average of two replicates of the air sampling each day.

**Figure 4:**
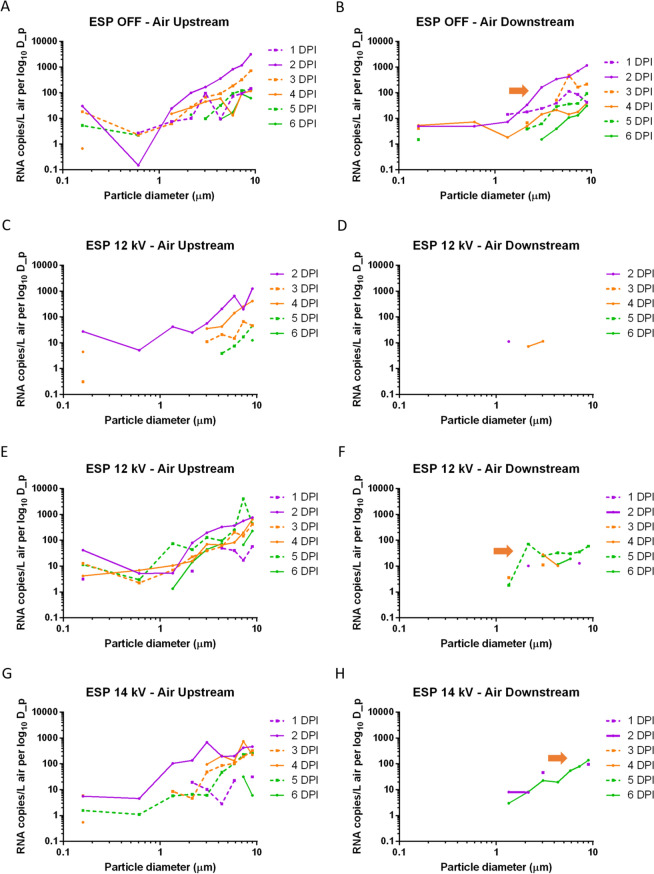
**Daily influenza A viral RNA size distribution measured using Andersen cascade impactors upstream and downstream of the electrostatic precipitator**. (**A**, **B**) ESP was off (positive control test); (**C**, **D**) ESP operated at 12 kV (1^st^ test); (**E**, **F**) ESP operated at 12 kV (2^nd^ test); (**G**, **H**) ESP operated at 14 kV (3^rd^ test). (**A**, **C**, **E**, **G**) Airborne viral RNA in upstream isolators; (**B**, **D**, **F**, **H**) airborne viral RNA in the downstream isolator. The Y axis represents RNA copies/m^3^ of the air normalized by the width of particle size interval. Orange arrows indicate the size distributions on the first day when at least one sentinel pig tested positive.

The size distribution of airborne IAV-laden particles collected by ACIs upstream and downstream each day are shown in Figure 4. In the positive control test, viral RNA was detected from multiple ACI stages both upstream and downstream in particle size ranges from 0.22 to > 8 μm. Interestingly, the highest viral load generated by the inoculated pigs measured upstream from 1 to 5 dpi was in particles > 8 μm with RNA concentrations ranging from 1.2 × 10^4^ RNA copies/m^3^ (4 dpi) to 3.1 × 10^5^ RNA copies/m^3^ (2 dpi), while the lowest viral load from 2 to 4 dpi was in particles between 0.22 μm and 1 μm (< 1.5 × 10^3^ RNA copies/m^3^). In the three ESP tests, viral RNA was detected in the upstream isolators across all size ranges for most of the sampling days. However, low concentrations of airborne IAV were detected from downstream samples mainly on particles 1 μm to 3.5 μm (all three ESP tests), 6.5 μm to 8 μm (2^nd^ test) and > 8 μm (3^rd^ test) before sentinel pigs tested positive, which indicated that the ESP decreased the airborne concentration of IAV across multiple size ranges. It is interesting to note in Figure 4 that no significant amount of RNA copies were found for particles in the sub-micrometer range, and the total RNA concentrations in the aerosol reported in Table 1 were mainly attributable to supermicrometer particles. We remark that we are not able to determine whether such particles are directly emitted from animal respiratory activities, or are aerosolized fomites/resuspended dust, as has been implicated in previous studies [33].

**Figure 5:**
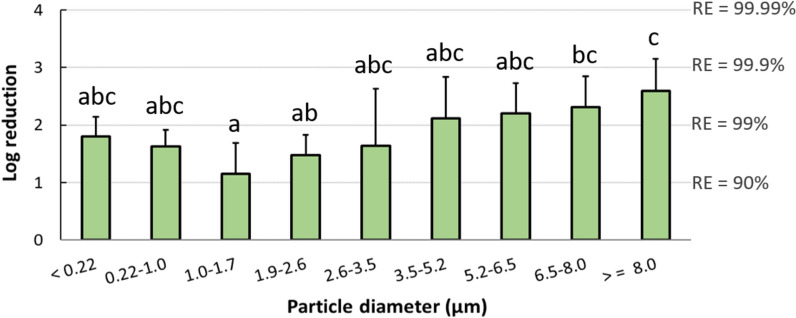
**Log reduction of airborne influenza A viral RNA concentrations by the ESP, as a function of particle size.** Log reduction was averaged over the three ESP tests and included all the days before sentinel pigs tested positive. Bars represent the means and error bars represent one standard deviation. Distinct letters above each bar are used to report significant differences resulting from multiple comparisons.

To quantify the airborne virus-laden particle removal by the ESP at different sizes, the average log reduction of airborne viral RNA concentrations in the three tests between upstream and downstream isolators was plotted for each particle size range (Figure 5). Data used for log reduction calculation corresponded to all the days before sentinel pigs tested positive, and data were not included when viral RNA was not detected in the upstream isolators. For the purpose of calculation, samples collected from downstream air and below the detection limit of IAV PCR were given a value of 50 copies/m^3^. The ESP decreased the concentration of IAV-laden particles emitted from inoculated pigs across all particle size ranges, with the higher removal efficiencies observed in particles > 6.5 μm and the lowest at 1.0–1.7 μm. While virus isolation was performed on all PCR-positive air samples, we were not able to recover viable IAV from any of the air samples collected by ACIs either upstream or downstream of the ESP.

In the case of PRRSV, total viral RNA copy concentrations in the air collected upstream and downstream of the ESP in each of the tests are shown in Table 2. In the positive control test, low levels of airborne viral RNA were detected from upstream and downstream isolators between 6 and 10 dpi, with a concentration ranging between 6.5 × 10^2^ and 4.3 × 10^5^ RNA copies/m^3^. In contrast, in the three ESP tests, almost no viral RNA was detected from the air either upstream or downstream of the ESP over the course of the tests.

**Table 2:** **Total airborne porcine reproductive and respiratory syndrome viral RNA concentration collected daily upstream and downstream of an electrostatic precipitator using Andersen cascade impactors**

		Airborne RNA copies/m^3^									
		1 dpi	2 dpi	3 dpi	4 dpi	5 dpi	6 dpi	7 dpi	8 dpi	9 dpi	10 dpi
ESP OFF	Upstream	ND	ND	ND	ND	ND	2.9E + 04	6.4E + 03	6.5E + 02	6.6E + 04	3.5E + 03
	Downstream	ND	ND	ND	ND	ND	9.3E + 03	9.9E + 03	4.3E + 05	2.3E + 04	ND
ESP 12 kV (1^st^ test)	Upstream	ND	ND	ND	ND	ND	ND	ND	ND	ND	ND
	Downstream	ND	ND	ND	ND	ND	ND	ND	ND	ND	ND
ESP 12 kV (2^nd^ test)	Upstream	ND	ND	ND	ND	ND	ND	ND	ND	ND	ND
	Downstream	ND	ND	ND	ND	ND	ND	ND	ND	ND	ND
ESP 14 kV (3^rd^ test)	Upstream	ND	ND	ND	ND	ND	1.6E + 03	ND	ND	NC	NC
	Downstream	ND	ND	ND	ND	ND	ND	ND	ND	NC	NC

Data shows the average of two replicates of the air sampling each day.

NC: not collected; ND: not detected.

### Quantification of viral load in environmental wipes

**Figure 6:**
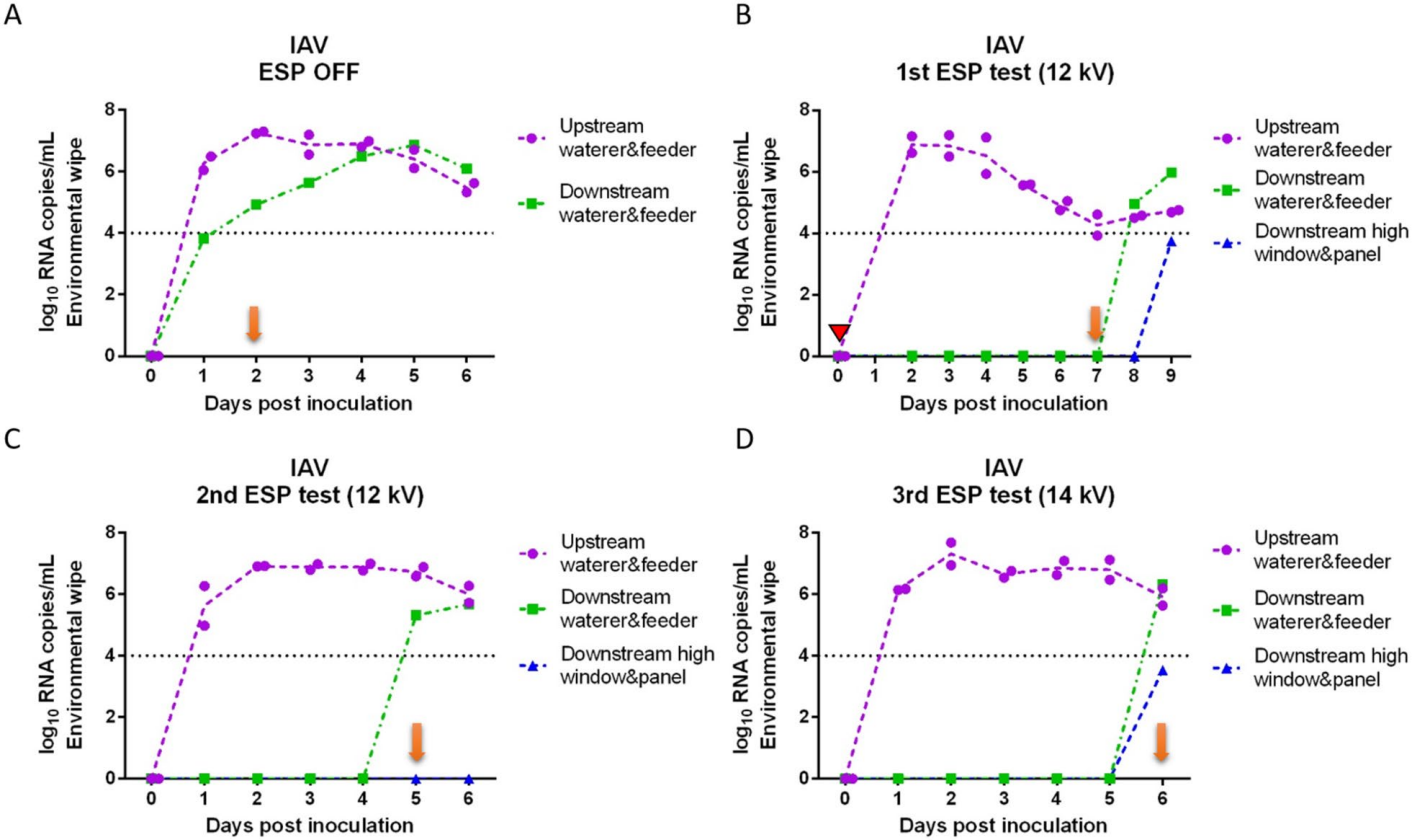
**Daily influenza A viral RNA concentration in environmental wipes collected inside isolators upstream and downstream of an electrostatic precipitator**. (**A**) ESP was off (positive control test); (**B**) ESP operated at 12 kV (1^st^ test); (**C**) ESP operated at 12 kV (2^nd^ test); (**D**) ESP operated at 14 kV (3^rd^ test). The red triangle indicates that the ESP was malfunctioning and the isolators were disconnected from the system. Orange arrows indicate the first day when at least one sentinel pig tested positive.

**Figure 7:**
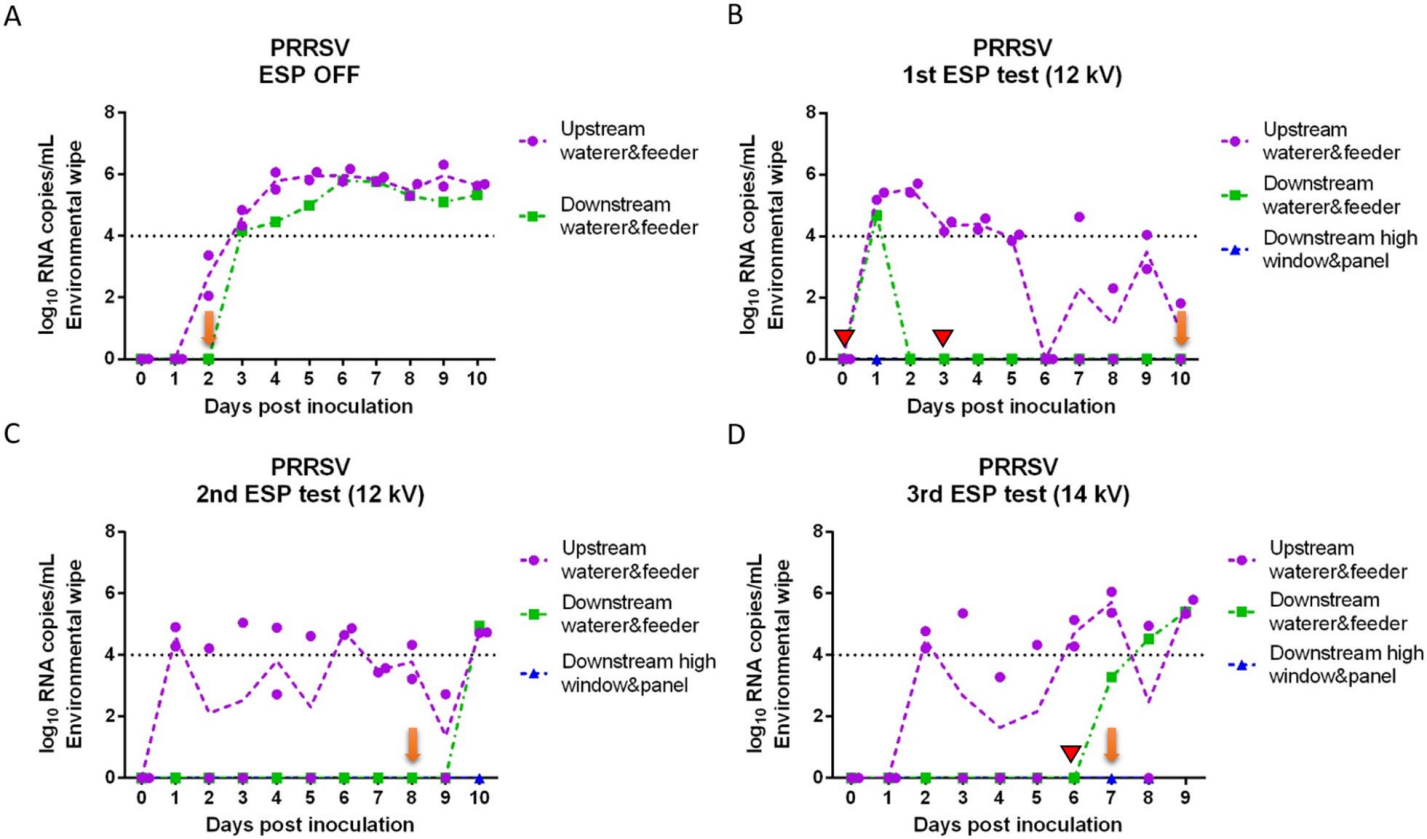
**Daily porcine reproductive and respiratory syndrome viral RNA concentration in environmental wipes collected inside isolators upstream and downstream of an electrostatic precipitator. **(**A**) ESP was off (positive control test); (**B**) ESP operated at 12 kV (1^st^ test); (**C**) ESP operated at 12 kV (2^nd^ test); (**D**) ESP operated at 14 kV (3^rd^ test). Red triangles indicate that the ESP was malfunctioning and the isolators had to be disconnected briefly (~1 day) during the 1^st^ ESP test, and that the ESP was shut down accidentally during the 3^rd^ test (~4 h). Orange arrows indicate the first day when at least one sentinel pig tested positive.

Viral RNA copy concentrations in environmental wipes collected daily from the surfaces of the feeder and waterer inside each isolator and the surfaces high for pigs to reach of the window and door inside the downstream isolator are shown in Figure 6 for IAV and Figure 7 for PRRSV. All the wipes collected from feeders and waterers from upstream isolators with IAV inoculated pigs tested positive from 1 dpi (or 2 dpi in the 2^nd^ ESP test) through 6 dpi (Figures 6A–D). In the positive control test, the wipes from the downstream waterer and feeder tested positive with a high Ct value (35.86) at 1 dpi which was one day before sentinel pigs tested positive. For the remaining of the tests with the ESP on, wipes of the downstream feeders and waterers usually tested positive on the same day (2^nd^ and 3^rd^ tests) or after sentinel pigs tested positive (1^st^ test). IAV was detected in the high part of the window and door inside the downstream isolator only on the same day (3^rd^ test) or after sentinel pigs became infected (1^st^ test).

In the case of PRRSV, there was noticeable variation in the RNA concentrations of wipes from the upstream isolators. Wipes of feeders and waterers from the upstream isolators with PRRSV inoculated pigs became positive at different days (1 to 3 dpi), and did not test consistently positive in the three ESP tests even though the pigs inside were infected. Generally, wipes from the downstream isolator tested positive after sentinel pigs tested positive.

## Discussion

Control of airborne transmitted diseases is especially relevant for livestock industries where animals are reared in confinement and in mechanically ventilated environments. In order to evaluate alternatives to air filtration to control the spread of airborne diseases between farms, we evaluated an ESP under experimental conditions on its ability to remove airborne virus-laden particles of two important swine viruses known to cause significant economic losses to the swine industry. We report significant reductions in the amount of virus-laden particles from the ESP-treated air which resulted in a delay in the transmission of the viruses to sentinel pigs. ESPs can be developed to have collection efficiencies above 99% across wide particle size ranges [20]. In our study, we used an ESP that generated positive ions by a series of narrow-diameter wires with a high voltage. The positively charged virus-laden particles subsequently deposited on grounded plates, resulting in the removal of a significant proportion of the particles from the airflow. In studies with intentionally aerosolized viruses, we also find evidence that in-flight inactivation may occur for viruses traveling close to the ESP wire electrodes, via exposure to short-lived reactive species in the corona discharge region [26]. Our study reported viral particle removal efficiencies by the ESP of 96.91% to > 99.97%, which is in line with those reported by other researchers [28, 30] and by our group where the same ESP was tested using generated viral aerosols in a wind tunnel [26].

Despite that the reduction in virus-laden particles from the air by the ESP could reach as high as 3 logs, infection of sentinel pigs happened in all the tests, which indicates that viable viruses were still transmitted through the air. However, we were not able to isolate viable viruses from any of the air samples. This may be due to the low sensitivity of cell culture methods to isolate viruses combined with the low concentration of the viruses in the air, the ventilation rate of 850 L min^−1^ that resulted in very high air exchange rates of 28.5 per hour in the isolators, or the air sampling methods. Isolation of viable viruses collected using similar methods to the ones of this study has been reported before while sampling artificial aerosols [41] or aerosols from infected pigs [42]. Alonso et al. [42] isolated IAV and PRRSV viruses from airborne particles larger than 2.1 μm generated by experimentally infected pigs. However, we operated the ACIs at 90 L/min instead of 30 L/min as in previous studies, which increases the possibility of detecting viral RNA from the air but may reduce the likelihood of virus viability due to impaction force. The delay of infection in sentinel pigs further confirmed that the ESP substantially removed viable viruses from the viral aerosols generated by the inoculated pigs, likely resulting in a lower airborne infectious dose to the sentinel pigs. Additionally, given that sentinel pigs had no immunity and we used a highly infectious strain [6], the infectious dose required to infect the sentinel pigs was presumably very low. The exposure time of sentinel pigs to the ESP-treated aerosols before sentinel pigs tested positive was similar among the ESP tests, suggesting that the infection of sentinel pigs was not related to unintentional contamination. The ESP working at a higher voltage of 14 kV delayed the IAV infection of sentinel pigs for one more day, which was expected, since the removal efficiency of the ESP is greater at higher voltage. In the third ESP test for PRRSV, the ESP shut down accidentally for 4 h in the early morning of 6 dpi, which likely resulted in transmission of infectious viruses as one sentinel pig tested positive in the next day. Overall, our results showed the potential of ESPs to reduce the transmission of airborne viruses.

The behavior of virus-laden particles in an aerosol is largely driven by particle size. In addition to calculating the removal efficiency of total airborne particles, we also estimated the removal efficiency of the ESP at different particle size ranges to explore whether certain sizes of particles were predominant in transmission of viruses to sentinel pigs. When the ESP was turned on, the ESP decreased the concentration of IAV-laden particles across multiple size ranges. However, low levels of viruses were detected in particles 1 μm to 3.5 μm and > 6.5 μm collected in the downstream isolator before sentinel pigs tested positive, indicating that the viruses responsible for transmission may be associated with particles in these specific size ranges. Nevertheless, additional studies are needed to further understand what virus-laden particle size ranges are implicated in disease transmission.

In the case of PRRSV, there were limited levels or even no PRRS viruses detected from the air collected upstream and downstream of the ESP. These results were surprising given that PRRSV is considered an airborne virus. A newly emerged PRRSV variant named L1C 1-4-4 was used for inoculation in this study. This variant appeared in late 2020 and resulted in massive PRRS outbreaks in the Midwestern US [5]. This variant was also deemed to have higher infectivity and potential transmissibility compared to other PRRSV-2 lineage 1 strains [6, 7]. However, there was no information on the airborne virus detection of this variant, even though airborne transmission was suspected due to the rapid regional spread of PRRS outbreaks [5]. Differences among PRRSV variants in their ability to transmit via an aerosol route have been documented [43–45]. Therefore, the size distribution, viral load and viability of airborne virus-laden particles generated by infected pigs may be different among different PRRSV strains. The limited detection of PRRSV L1C 1-4-4 variant in the air may be due to viruses being loaded to particles with sizes outside the ranges collected by ACIs, or the viral load released into the air was too low to be detected by ACIs under the conditions of this study where the airflow may remove the low concentrations of airborne viruses too fast from the air stream. Alternatively, a less likely explanation but nevertheless possible, is that infection with IAV may have activated an innate immune response that was somewhat protective against PRRSV infection [46, 47]. Even though the challenged pigs were infected with PRRSV, the amount of PRRSV shed through secretions may have been lower and not able to be detected from air samples. Additional tests using pigs without prior known viral infections challenged and inoculated with other PRRSV strains, such as MN-184 which has been reported in the aerosol from infected pigs [42, 43], would offer an opportunity to better quantify the PRRSV removal efficiency of the ESP.

We used environmental sampling as another approach to document transport of viral aerosols. We targeted sampling of the surfaces of feeders, waterers, windows and doors inside each isolator. When the ESP was turned off, wipes from the downstream isolator showed evidence that IAV was being transported through the air and was able to contaminate the environment within one day post-inoculation of the challenged pigs and before sentinel pigs tested positive. However, when the ESP was turned on, wipes from the downstream isolator only tested positive on the same day or after sentinel pigs tested positive, suggesting that the source of environmental contamination was likely from the sentinel pigs after they became infected. These results provided evidence that the ESP was not only able to remove particles suspended in the air but also decreased the viral load depositing on surfaces. We also acknowledge that the overall sensitivity of surface sampling to identify viral aerosol transport is general low.

During this study, we found that relative humidity (RH) played an important role on the working stability of the ESP, with the ESP being more stable when it operated at low RH. Changes in the RH of the environment may affect the magnitude of the electric current generated by the corona discharge [48]. Therefore, we used a dehumidifier to keep the RH inside the isolators within 40% to 60% for most of the time. However, we had to open the isolators daily to sample and feed the pigs, which resulted in an increase of the RH inside the isolators and an unstable voltage of the ESP at certain times. These changes of the conditions may have had an effect on the removal efficiency of the ESP during that time. The RH may have also affected the transport and the viability of virus-laden particles [49]. Besides, the ESPs are found to have higher removal efficiencies when operating at lower air flows since particles are more easily ionized when they migrate more slowly through the ESP. In this study, we only tested the ESP working at one air flow of 850 L min^−1^ (~28.5 air changes per hour in the isolator system) which is quite high for conditions found in indoor environments. Lower air flows may have resulted in higher removal of virus-laden particles by the ESP and perhaps would have fully prevented transmission of the viruses to the pigs. Further studies to explore the role of RH and air flow on the removal efficiency and working stability and safety of the ESP under field conditions are necessary.

In summary, under the conditions of this study, the ESP designed as part of this study was able to efficiently decrease the concentration of airborne viruses from infectious aerosols generated by infected pigs. Our results support the potential for ESPs to prevent the spread of airborne viruses in animal farming facilities. Along with minimum pressure drop and potential lower energy consumption, ESPs may be a promising alternative to air filtration systems. Further research is needed to evaluate ESPs under field conditions to fully ascertain the potential of this technology to prevent the spread of airborne viruses between farms.

## Data Availability

All data generated or analyzed during this study are included in this article.
